# Meningococcal Septic Shock Revealed: The Frightening Face of Serogroup B

**DOI:** 10.7759/cureus.89950

**Published:** 2025-08-12

**Authors:** Belkis Hatice Inceli, Döndü Nilay Penezoglu, Merve Havan, Selin Nar Otgun, Ebru Evren, Zeynep Ceren Karahan, Tanıl Kendirli, Halil Özdemir, Ergin Çiftçi

**Affiliations:** 1 Pediatric Infectious Diseases, Ankara University School of Medicine, Ankara, TUR; 2 Infectious Disease, Ankara University School of Medicine, Ankara, TUR; 3 Intensive Care, Ankara University School of Medicine, Ankara, TUR; 4 Infectious Disease, Microbiology, Public Health Agency of Turkey, Ankara, TUR; 5 Microbiology, Ankara University School of Medicine, Ankara, TUR; 6 Pediatric Intensive Care, Ankara University School of Medicine, Ankara, TUR

**Keywords:** meningococcemia, molecular diagnosis, neisseria meningitidis serogroup b, purpura fulminans, septic shock

## Abstract

Meningococcal infections are still a serious cause of morbidity and mortality in children, despite vaccine developments or advancements in vaccination, antibiotics, supportive treatments, and advancements in intensive care facilities.* Neisseria meningitidis *has 12 serogroups, among which A, B, C, Y, and W are responsible for the most invasive infections. Sudden onset, rapid worsening, and high contagiousness are important clinical features of meningococcal infections. Purpura fulminans, septic shock, multiple organ failure, and increased intracranial pressure also constitute life-threatening complications. Here, we present a six-month-old female patient who presented with complaints of fever, malaise, and rash and was diagnosed with meningococcal sepsis and shock based on clinical examination, laboratory findings, and blood polymerase chain reaction (PCR) positivity. She was treated in the pediatric intensive care unit (PICU) but died despite the treatments.

## Introduction

*Neisseria meningitidis* (meningococci) is a Gram-negative encapsulated bacterium that causes invasive meningococcal disease (IMD). Its only known host is humans, and it is transmitted from one human to another via droplets or nasopharyngeal secretions [1,2]. The incidence of IMD is particularly high in infants and children below the age of five, and its rate of mortality is about 10-15%. In addition, IMD is associated with long-term physical, neurological, and psychological sequelae. These sequelae include hearing loss, mental retardation, extremity amputations, seizures, cognitive disorders, reduced learning and memory skills, and anxiety disorder [3].

The pathogenesis of IMD is directly linked with the capsule of the bacterium, and the capsule allows the bacteria to be carried to several parts of the body, including the central nervous system (meningitis) via blood circulation, by preventing opsonophagocytosis [4]. Based on variations in the chemical composition of the bacterial polysaccharide capsule, 12 different serogroups with various epidemiological characteristics, such as prevalence, virulence, immunogenicity, and geographical and temporal distribution, have been identified (A, B, C, E, H, I, K, L, W, X, Y, and Z). Five of these 12 serogroups (A, B, C, W, and Y) have historically caused most IMD cases, and they have been associated with dynamic and unpredictable epidemiology differing based on time and region [5,6]. Historically, serogroup A and, less prevalently, serogroup C have been the main causes of large-scale epidemics and pandemics, especially in Africa, Asia, and South America. Moreover, serogroup B poses a relatively more recent sporadic, endemic, and rampant threat in North America, Europe, South America, and Oceania [7]. It is important to identify the prevalent serogroups in planning a vaccination program and management strategy because the distribution of prevalent serogroups may vary depending on countries and other factors. According to surveillance studies conducted between 2005 and 2018, the most common causative agent of bacterial meningitis in Turkey is *N. meningitidis*, and the most common strain is MenB. The current national immunization schedule in Turkey does not include meningococcal vaccination. Men A, B, C, and W vaccines and Men B vaccine are licensed in Turkey and can be easily accessed by families who wish to receive this vaccine [8]. Due to the higher mortality and morbidity rates of invasive meningococcal infections, appropriate molecular epidemiological analyses are needed to manage and control the spread of the disease. In this case report, to emphasize the significance of IMD, we present a six-month-old female patient who was treated in the pediatric intensive care unit (PICU) due to septic shock secondary to IMD caused by serogroup B, which was determined only blood PCR method, but died despite the treatments.

## Case presentation

The six-month-old female patient was brought to our hospital with complaints of fever, malaise, and rashes. The fever was acute in onset, the patient refused to be breastfed, and became weak. She also developed rashes that started on her legs and then spread quickly to the rest of her body. The rash started as petechiae and turned into purpura fulminans within a few hours.

**Figure 1:**
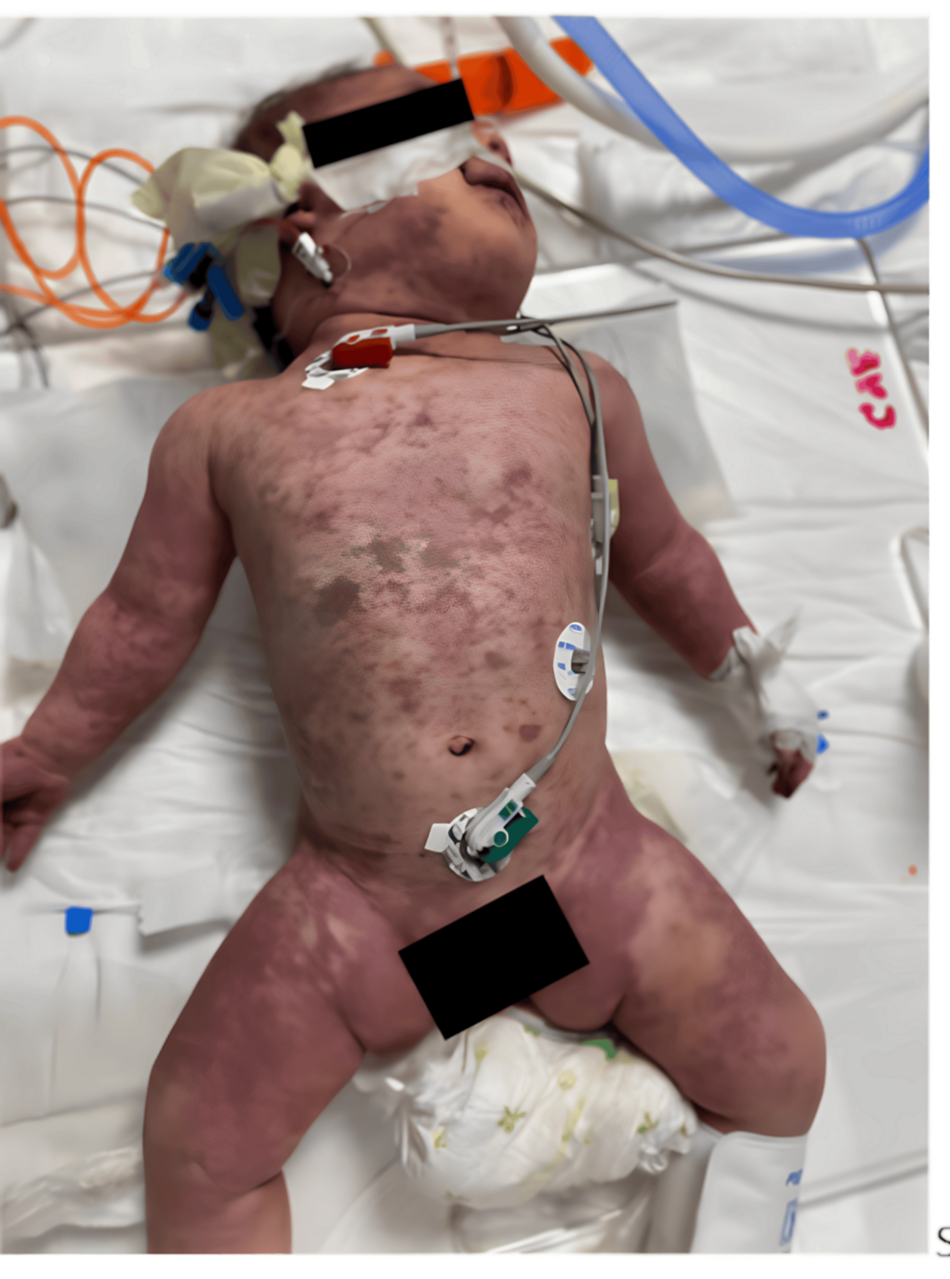
Widespread petechial and purpuric rashes on the patient's body

Based on her history, it was learned that the patient’s parents were third-degree relatives; she was born at term by cesarean section with a birthweight of 3,500 g; she was being breastfed; she had received all applicable vaccines, except for meningococcal vaccines; and her neuromotor development was suitable for her age. On physical examination, the patient had a poor general state, her body temperature was 35.2°C, peak heart rate was 80/min, respiratory rate was 40/min, blood pressure was too low for measurement, hands and feet were cold, central and peripheral pulse was weak, and capillary refill time was 7 seconds. The liver was palpable at 4 cm under the rib, and there were petechial and purpuric rashes on her entire body, which had a tendency to combine and did not blanch on pressure (Figure 1). The patient was taken to the isolation room in the PICU with a pre-diagnosis of meningococcemia and septic shock. While the presentation is classic, we have ruled out differential diagnoses (e.g., viral exanthema, other causes of sepsis with rash such as Strep pyogenes, Rickettsia, etc.). The patient, who was started on 20 mL/kg normal saline infusion and 100 mg/kg/day ceftriaxone in two doses, was connected to the mechanical ventilator following rapid sequence endotracheal intubation when her breathing became shallow. Because her low blood pressure persisted, two bolus treatments of 0.9% NaCl at 20 mL/kg were administered, and central vein and artery catheters were applied. The blood tests of the patient revealed anemia (Hb: 7.3 g/dL), thrombocytopenia (platelets: 8,000/mm^3^), neutropenia (WBC: 4,630/mm^3^, total neutrophil count: 660/mm^3^) in CBC, hypoglycemia (glucose: 6 mg/dL), hypoproteinemia and hypoalbuminemia (total protein: 16.9 g/L, albumin 10.9 g/L), hypocalcemia (calcium: 6 mg/dL), hyperphosphatemia, and hyperkalemia (P: 7.95 mg/dL, K: 7.2 mmol/L) in biochemistry, and severe acidosis (pH: 7.05, pCO_2_: 44.1 mmHg, pO_2_: 31.9 mmHg, HCO_3_: 10.4 mEq/L, lactate: 12.1 mmol/L) in blood gases. Her CRP value was 57.2 mg/L, while her coagulation parameters were too long for measurement (Table 1).

**Table 1:** Laboratory results of the patients BNP: brain natriuretic peptide; CRP: C-reactive protein; ALT: alanine transaminase; APTT: activated partial thromboplastin time; AST: aspartate transaminase; INR: international normalized ratio; pCO₂: partial pressure of carbon dioxide; pO₂: partial pressure of oxygen; HCO₃: sodium bicarbonate; Hb: hemoglobin; PT: prothrombin time

Test Category	Parameter	Result	Reference Range
Complete Blood Count	Hb (g/dL)	7.3	11.5-15
Complete Blood Count	White Blood Cell (mm³)	4630	5-13.5
Complete Blood Count	Total Neutrophil Count (mm³)	660	1.5-8
Complete Blood Count	Total Lymphocyte Count (mm³)	3150	1.5-7
Complete Blood Count	Platelet (mm³)	8000	150-450
Complete Blood Count	CRP (mg/L)	57.2	0-5
Biochemistry	Glucose (mg/dL)	6	74-100
Biochemistry	Urea (mg/dL)	30	10.5-38.5
Biochemistry	Creatinine (mg/dL)	0.49	0.7-1.20
Biochemistry	Na (mmol/L)	142	136-145
Biochemistry	K (mmol/L)	7.2	3.5-5.5
Biochemistry	Total Protein (g/L)	16.9	60-80
Biochemistry	Albumin (g/L)	10.9	38-54
Biochemistry	Ca (mg/dL)	6	9.5-10.5
Biochemistry	P (mg/dL)	7.95	2.5-4.5
Biochemistry	AST (U/L)	99	0-50
Biochemistry	ALT (U/L)	36	0-50
Coagulation Tests	APTT (sec)	Unmeasurably Long	25-36.5
Coagulation Tests	PT (sec)	Unmeasurably Long	9.5-12.5
Coagulation Tests	INR	Unmeasurably Long	0.83-1.11
Coagulation Tests	BNP (pg/mL)	2264	0-125
Coagulation Tests	D-Dimer (ng/mL)	4750	0-125
Blood Gas	pH	7.05	-
Blood Gas	pCO₂	44.1	-
Blood Gas	pO₂	31.9	-
Blood Gas	HCO₃	10.7	-
Blood Gas	Lactate	12.1	-

NaHCO_3_ infusion for the severe metabolic acidosis of the patient, 10% Ca-gluconate for her hypocalcemia, fluid treatment with dextrose at a suitable ratio for her hypoglycemia, albumin infusion for her hypoalbuminemia, and vitamin K and fresh frozen plasma treatment for her coagulation dysfunction were started. Because the patient’s hypotension persisted despite fluid treatment, epinephrine (0.1 µcg/kg/min), and norepinephrine (0.1 µcg/kg/min), their doses increased to 1 mcg/kg/min. Promptly added hydrocortisone (100 mg/m²/day) for possible adrenal failure was given consecutively. The patient was supplemented with blood products for anemia and bleeding dysfunction and was planned to be kept in venoarterial extracorporeal membrane oxygenation (ECMO). However, she developed cardiac arrest in the 15th hour of admission and could not be saved despite all the efforts and interventions.

For further analysis, the serum sample collected (around the same time the patient was tried to be revived) was sent to the National Reference Laboratory for Respiratory Pathogens under the Division of Microbiology Reference Laboratories and Biological Products affiliated with the General Directorate of Public Health. Nucleic acid extraction and purification procedures were carried out on the clinical samples using a Nucleic Acid Isolation System (Zybio EXM 3000) and a Rapid Nucleic Acid Extraction Kit (Bio-Speedy, Turkey). The presence of invasive bacterial pathogens in the purified nucleic acid extracts was analyzed using the real-time PCR method. Using a *Haemophilus influenzae, Neisseria meningitidis*, and *Streptococcus pneumoniae *qPCR kit (Bio-Speedy, Turkey), *N. meningitidis* sodC genes were identified in serum samples. After the identification of *N. meningitidis *in the purified nucleic acid extracts obtained from the clinical samples, to determine meningococcal serogroups, the real-time PCR method was utilized to screen the samples for molecular serogroups A, B, C, W, X, and Y. As a result, it was determined that the samples contained serogroup B-specific gene regions.

Consequently, the samples were first diagnosed with *N. meningitidis* and then identified to have *N. meningitidis *serogroup B in the next stage using two different real-time PCR methods. Because the patient was diagnosed with meningococcemia, chemoprophylaxis with ciprofloxacin or ceftriaxone was started for the healthcare personnel who were in contact with the patient, the patient’s family members who were in close contact with her, and other individuals who traveled with the patient for at least eight hours.

## Discussion

IMD is a systemic infection caused by the Gram-negative encapsulated bacterium *N. meningitidis*. While this pathogen is usually colonized in the human nasopharynx (less frequently in the urogenital system and anal canal) and can be isolated from approximately 10% of individuals, invasive disease is rare [9]. Nevertheless, it is a serious and rapidly progressing condition that has a mortality rate of 6-8% despite appropriate interventions, a high risk of sequelae, and is associated with life-threatening sepsis [10]. The global disease burden of IMD has continued for a long time at a significant and unpredictable degree. IMD can be seen in all age groups, while it is most frequently seen in infants below one year of age, followed by the one to four age group. In some countries, it has a secondary peak in adolescents and young adults (15-24 years old) [11,12]. It is believed that the increasing socialization and behavioral changes in this age group are responsible for this secondary peak. Living in crowded spaces, physical proximity, second-hand smoke, and recent respiratory tract infections were identified as significant risk factors for IMD in children and adolescents [13]. Factors associated with hospitalization due to IMD include low household income levels, in addition to medical conditions, such as immunodeficiency, asplenia/hyposplenism, autoimmune diseases, hemophilia, and severe chronic respiratory disorders [14].

The most prevalent clinical form of IMD is meningitis, which is seen in approximately 60% of infected patients. In 10-20% of cases, fulminant sepsis or shock develops with or without meningitis. At the time of presentation, 30% of cases display only fever and rashes without signs of meningitis or shock. Although the bacterial load, endotoxin concentrations, and inflammatory mediators of these patients are low, meningitis and sepsis can develop if they are not treated in a timely manner. Meningococcal sepsis is a clinical condition that must be diagnosed fast because it progresses rapidly, and the early start of treatment with early diagnosis and the appropriate antibiotic agent affects the prognosis. As signs and symptoms are non-specific at the onset of the disease, it is difficult to make a clear differential diagnosis [13,14]. In 2008, Theilen et al. summarized the guidelines of the Scottish Intercollegiate Guidelines Network regarding the treatment of invasive meningococcal infections and contributed them to the literature. In the guidelines, it was emphasized that meningococcal infection should be excluded not immediately, but within four to six hours in patients with symptoms of nausea, vomiting, fever, malaise, malnutrition, and irritability; rashes should be checked during follow-up. Invasive meningococcal infection must be considered in children with a toxic clinical picture, especially when accompanied by headache, changes in consciousness, neck stiffness, and petechial rashes [15].

Rashes are among the most significant signs of IMD. While they are usually purpuric or petechial, maculopapular rashes may also be seen. Hemorrhagic skin lesions are present in 28-77% of patients at the time of presentation. Therefore, meningococcal disease can emerge even in the absence of petechiae and other hemorrhagic skin lesions. It was reported that *N. meningitidis* is responsible for 92% of cases showing rashes. Rashes can also accompany meningococcal sepsis [6,16,17]. Our patient also had petechial and purpuric rashes that were likely to combine and did not blanch under pressure in her entire body when she was brought to our hospital.

Based on differences in the chemical composition of the bacterial polysaccharide capsule, which determines the virulence of *N. meningitidis*, 12 meningococcal serogroups have been defined: A, B, C, E, H, I, K, L, W, X, Y, and Z. Five of these 12 serogroups (A, B, C, W, and Y) have historically caused most IMD cases, and they have been associated with dynamic and unpredictable epidemiology differing based on time and region [18]. The first conjugate meningococcal vaccine was introduced in 1999 in the United Kingdom against serogroup C, followed by four-valent (serogroups A, C, Y, and W) and one-valent (serogroup A) vaccines licensed in 2000 and 2010, respectively. In January 2013, a four-valent meningitis B vaccine (4CMenB) containing four main immunogenic components and targeting the PorA serosubtype P1.4.5, 6 via the New Zealand NZ98/254 strain OMV (ZN OMV) was approved by the European Commission and later licensed in Canada and Australia [19]. The 4CMenB vaccine was introduced in Türkiye after it was licensed in November 2018.

In a review study conducted to investigate the global epidemiology of IMD between 2010 and 2019, 90 surveillance reports and 22 articles on the epidemiology of IMD in 77 countries were examined. The highest incidence values were observed in countries in the African meningitis belt, while other countries, such as New Zealand, Ireland, Lithuania, and the United Kingdom, also showed high rates of incidence. It was determined that serogroup B constituted the highest percentage of classifiable IMD cases in Australia, New Zealand, and many countries in Europe and the Americas, and among all age groups, infants and small children were mainly affected by serogroup B [13].

In an epidemiological study carried out with 278 cases series in 2011-2020 in the Netherlands, 31.3-67% of children with IMD were admitted to intensive care services, 37% were put under mechanical ventilation, 30% required inotropic support, 18% developed multi-organ failure, and mortalities were reported in 4.1-9.9%. Serogroup B was isolated as the cause in 55% of patients, and it was shown that presumably 73% of these isolates were within the scope of the 4CMenB vaccine. The 4CMenB vaccine was not included in the national vaccination plan of the Netherlands, but it was concluded that the number of IMD cases would substantially drop if it came into use [20].

IMD is sporadic in general. The most well-defined risk factor for meningococcal disease development is close contact with a primary case. People living in the same home, school, or common area can lead to case clusters and outbreaks. The risk of IMD increases particularly in the contacts of the index case at their home. Although the risk of IMD in family members continues within 14-365 days following contact, according to Hoek et al., most infections develop within the first 14 days. The rate of attacks in those in contact at home who are receiving chemoprophylaxis is 1.1/1000 [21]. If chemoprophylaxis is not administered, the risk of IMD among those in contact at home is 1,000 times higher compared to the general population. It is highly important to start chemoprophylaxis within 24 hours after presentation in those who are in close contact with the case, rather than waiting for confirmation by culture and serotyping. In the case report by Bayhan et al. about twin infants diagnosed with meningitis in connection with *N. meningitidis* serogroup B two days apart, it was stated that, when fever started in Case 2, Case 1 was not given chemoprophylaxis because test results were not available. However, prophylaxis was prescribed to others in close contact right after meningococcal test results, and no other case of meningococcal disease was seen in the family [22].

The four-component protein-based meningococcal B vaccine (4CMenB; Bexsero, GSK) was licensed in the European Union in 2013. In a study in Spain examining vaccine efficacy in IMD cases younger than 60 months old diagnosed between 5 October 2015 and 6 October 2019, full vaccination with 4CMenB was found to be 76% effective in preventing meningococcal disease caused by any serogroup, 71% effective in preventing disease caused by serogroup B, and 92% effective in preventing disease caused by meningococci other than serogroup B. Additionally, having at least one dose of vaccination with 4CMenB was shown to have a moderate preventive effect (54%) in cases where vaccination plans could not be completed due to young age or lack of vaccines [23]. Full vaccination with 4CMenB was determined to be effective in preventing invasive disease caused by serogroup B meningococci and non-serogroup B meningococci in children under the age of five.

Today, invasive bacterial diseases caused by *N. meningitidis*, *S. pneumoniae*, and *H. influenzae* type b (meningitis, sepsis, and pneumonia accompanied by bacteremia) are surveyed by the Turkish Ministry of Health within the scope of the “Directive for the Surveillance of Vaccine-Preventable Invasive Bacterial Diseases: 2016/23.” According to the legislation in Türkiye, IMD cases are among notifiable communicable diseases [24].

Molecular diagnosis and typing methods were developed in the Ministry of Development project on the “Development of Laboratory Capacity for the Molecular Diagnosis and Typing of Notifiable Communicable Diseases Surveyed under Specific Disease Control Programs in Türkiye” implemented by the National Microbiology Reference Laboratories of the General Directorate of Public Health. Using the real-time PCR method with high diagnostic accuracy, sensitivity, and specificity developed in the project, *N. meningitidis*, *S. pneumoniae*, and *H. influenzae* type b can be detected and serotyped within a few hours using clinical samples collected from cases with meningitis, bacteremia, or sepsis such as CSF, serum, and petechiae/purpura. The method that was developed and validated for the identification of these three pathogens received an ISO 15189 accreditation certificate from the Turkish Accreditation Agency (TÜRKAK).

Prior to the development of PCR techniques, the microbiological diagnosis of meningococcal infections typically relied on the cultivation or visualization of Neisseria meningitidis from sterile body sites (e.g., blood, cerebrospinal fluid (CSF), or petechiae), the detection of capsular polysaccharide in the CSF, or the identification of a significant antibody response several weeks after acute infection. The incorporation of PCR into diagnostic tools represented a major advancement in the diagnosis of systemic meningococcal disease [25].

In a study by Øvstebø et al. [26], neisserial DNA was quantified in the plasma of 65 patients and in the CSF of 12 patients with systemic meningococcal disease. The authors demonstrated that, unlike conventional blood cultures, quantitative PCR enabled the estimation of the total meningococcal load in plasma and allowed for the prediction of disease severity and clinical outcomes during hospitalization [26].

It is expected that the current laboratory studies will help the development of new vaccine formulations that are more effective for the Turkish population and the creation of vaccination policies.

## Conclusions

Consequently, meningococcal infections are still a serious cause of morbidity and mortality in children, despite advancements in vaccination, antibiotics, supportive care treatment, and intensive care facilities. They also create a continued burden on children, families, medical resources, and, thus, the economy. The preferred option for the control of MenB may involve routine infant vaccination, catch-up vaccination in small children, and the direct protection of adolescents by routine vaccination. Considering that serogroups may vary, it is needed to highlight the importance of administering vaccines that have been developed against IMD caused by serogroups A, C, W, and Y. In this case report, we want to should focus on emphasizing that the mortality due to *N. meningitidis-induced* septic shock is still very high and can lead to death within hours in a previously healthy patient, as well as the role of early recognition, early diagnostic testing, adherence to septic shock protocols, vaccination, and infection prevention. Purpuric rash in an infant warrants immediate evaluation for meningococcemia. Do not delay antibiotic initiation pending lab confirmation.
